# Analogue pattern recognition with stochastic switching binary CMOS-integrated memristive devices

**DOI:** 10.1038/s41598-020-71334-x

**Published:** 2020-09-02

**Authors:** Finn Zahari, Eduardo Pérez, Mamathamba Kalishettyhalli Mahadevaiah, Hermann Kohlstedt, Christian Wenger, Martin Ziegler

**Affiliations:** 1 Nanoelektronik, Technische Fakultät, Christian-Albrechts-Universität zu Kiel, 24143 Kiel, Germany; 2 IHP – Leibniz-Institut für innovative Mikroelektronik, 15236 Frankfurt (Oder), Germany; 3 BTU Cottbus-Senftenberg, 03046 Cottbus, Germany; 4 Mikro- und nanoelektronische Systeme, Fakultät für Elektrotechnik und Informationstechnik, Technische Universität Ilmenau, 98693 Ilmenau, Germany

**Keywords:** Electrical and electronic engineering, Electronic devices, Information storage, Learning algorithms, Machine learning

## Abstract

Biological neural networks outperform current computer technology in terms of power consumption and computing speed while performing associative tasks, such as pattern recognition. The analogue and massive parallel in-memory computing in biology differs strongly from conventional transistor electronics that rely on the von Neumann architecture. Therefore, novel bio-inspired computing architectures have been attracting a lot of attention in the field of neuromorphic computing. Here, memristive devices, which serve as non-volatile resistive memory, are employed to emulate the plastic behaviour of biological synapses. In particular, CMOS integrated resistive random access memory (RRAM) devices are promising candidates to extend conventional CMOS technology to neuromorphic systems. However, dealing with the inherent stochasticity of resistive switching can be challenging for network performance. In this work, the probabilistic switching is exploited to emulate stochastic plasticity with fully CMOS integrated binary RRAM devices. Two different RRAM technologies with different device variabilities are investigated in detail, and their potential applications in stochastic artificial neural networks (StochANNs) capable of solving MNIST pattern recognition tasks is examined. A mixed-signal implementation with hardware synapses and software neurons combined with numerical simulations shows that the proposed concept of stochastic computing is able to process analogue data with binary memory cells.

## Introduction

The use of memristive devices in neuromorphic electronics has experienced a rapid upswing in recent years^1–3^. This growing interest is due to the wider use of machine learning methods and the associated steadily increasing energy requirements of today’s technologies. Neuromorphic electronics^4^ do not rely on the von Neumann architecture, known to limit processing time and lead to a high energy consumption^5–7^. Therefore, they provide a promising way to overcome the physical and economical limits that will soon be reached by complementary metal-oxide semiconductor (CMOS) technology^8–10^.

In addition, neuromorphic technology can be exploited to develop novel computing architectures inspired from biological systems^1,11–15^. In biology, neurons composing the nervous system are interconnected by synapses whose plastic coupling strengths can change due to specific neural activity patterns^16^. This behaviour is referred to as synaptic plasticity and its important features—long-term potentiation (LTP) and its counterpart long-term depression (LTD)—have been identified as fundamental mechanisms responsible for learning and memory in nature^17^. Furthermore, the relative timing of the activities of the pre- and post-synaptic neurons, known as spike-timing dependent plasticity (STDP), is crucial to the induction of LTP or LTD^18^. Moreover, as biological systems show outstanding performances in dealing with noisy data, it is possible to reproduce their biological information processing using probabilistic models^19–22^.

The aforementioned nervous system behaviour can be emulated with neuromorphic hardware based on CMOS technology^23–26^. In particular, combining these networks with memristive devices has the potential to reproduce synaptic plasticity through the devices’ ability to perform non-volatile resistance change^2,11,27^. Several types of devices show memristive effects and exploit a wide variety of material systems. Various physical mechanisms can be responsible for the switching resistance^1,3,28^. In state-of-the-art neuromorphic systems, memristive devices are mainly used for vector matrix multiplications in hardware accelerators to train deep neural networks (DNNs). This allows for a tremendous decrease of energy demand and training time^29–31^. However, the device performance needs to meet very strict requirements regarding the number of states, switching symmetry, or variability to compare with network performances reached with weights in floating point precision^32,33^.

While DNNs present a powerful approach, biological systems can be more accurately reproduced using spiking neural networks (SNNs)^14,33^. These approaches often employ unsupervised learning schemes and treat a very large amount of unlabelled data^34–38^. SNNs necessitate lower device requirements and are less sensitive to device variability than DNNs. In this context, stochastic neural networks are particularly interesting as they exploit the inherent stochastic nature of resistive switching. Here, the variability of memristive devices is used explicitly for the technical implementation of biological information processing. Such stochastic systems include noise tolerant stochastic computing technologies^39^, synchronisation of oscillatory neurons to emulate neuronal coherence^40,41^, stochastic switching neurons^42–45^ and stochastic learning rules realised with single binary synapses^46–51^, as well as compounds of several binary devices as one synapse^42,46,48,49,52^.

In binary resistive random access memory (RRAM) devices, the resistance changes between a high resistance state (HRS) and a low resistance state (LRS) through the formation and dissolution of conductive filaments within an insulating layer. Since the switching process has an inherent stochastic nature, such memristive devices are promising for stochastic neural networks^39,46^. Furthermore, RRAM devices are already integrated in CMOS technologies^53,54^. While the potential of RRAM cells for stochastic learning was recently shown^47^, a thorough investigation of the concept has yet to be reported. In particular, the influence of the memristive cells’ important technological parameters on their performance in stochastic neural networks has been given very little attention. Furthermore, the ability of networks utilising multilevel and analogue devices^31,34–37,55^ to complete similar tasks must be compared to verify the long term viability of stochastic learning based on binary RRAM cells.

This work investigates a stochastic artificial neural network (StochANN) based on fully CMOS integrated binary RRAM devices^54,56,57^. While the experimental realisation of the network composed of hardware synapses, i.e. stochastic binary RRAM devices based on polycrystalline HfO_2-x_, and software neurons has already been reported^47^, a detailed description of the developed learning algorithm is given here for the first time. Moreover, the experimental results on recognising different patterns from the MNIST^58^ benchmark dataset are compared to results obtained in numerical simulations of the network for the first time. Two different RRAM technologies, namely devices based on polycrystalline and amorphous HfO_2−x,_ are compared on the device level as well as on the system level regarding the capability of emulating the proposed stochastic binary synapses in experimental realisations of the StochANN. The impact of different intrinsic switching variabilities on stochastic learning is studied in detail. In addition, the characterisation of both device technologies provided in^57^ are extended in terms of switching probability and retention. Furthermore, the algorithm is extended in this work to considerably increase the recognition accuracy for the whole MNIST dataset as it is shown in simulation results. Based on the experiments and simulations, we show evidence that the proposed concept of stochastic computing is able to process analogue information with binary memory cells. This offers an interesting alternative to concepts that exploit multilevel resistance states for neuromorphic computing^57^.

The manuscript is organised as follows: In the “StochANN - stochastic artificial neural network” section the emulation of the stochastic synaptic plasticity is explained, followed by a description of the two-layer feed forward network and the learning algorithm employed here, which is utilizing a local stochastic update rule emulating LTP and LTD inspired by biological neural networks. The devices’ variabilities are then analysed in terms of endurance, yield, and retention. In the “Results” section, the performance of the experimental network is assessed in terms of recognition accuracy, and the impact of the different switching variabilities on the performance is discussed. The “Discussion” section includes simulations of the experimental network as well as simulations of larger networks. These larger networks are compared to the recognition performance of similar network structures and to that of deep neural networks provided in the literature.

## StochANN - stochastic artificial neural network

### Emulation of synaptic plasticity

The inherent randomness of switching RRAM devices is employed as a plasticity model according to Ref.^47^. Fully CMOS-integrated 4 kbit RRAM arrays are used in a 1-transistor-1-resistor (1T-1R) configuration^54,56^. These cells are layered with TiN as bottom electrode, HfO_2−x_/TiO_2−y_ bilayer as active layers, and TiN as top electrode. The devices possess binary resistance states, i.e. an HRS and a LRS. Before operating the devices, an effective electroforming step is required. Therefore, the incremental step pulse with verify algorithm (ISPVA)^59,60^ is used. Resistive switching occurs stochastically through the formation and rupturing of conductive filaments consisting of oxygen vacancies^56,61^. The switching to LRS, i.e. the formation of the conductive filament, is caused by the hopping of charged vacancies, which are then reduced at the filament, thereby becoming immobile^61^. The dissolution of the filament leads to switching to the HRS and is achieved by applying a voltage of opposite polarity. Joule heating and the electric field lead to oxidation of the vacancies and a subsequent drift of the charged vacancies. As a result, the diameter of the filament is thinned out and the filament gets ruptured^61^. In Ref.^62^ the reset process is also discussed in terms of thermo-electrochemical effects of Joule heating and ion mobility. The reset transition proceeds in gradual resistance changes, covering a limited resistance window.

According to Ref.^40,47^, the switching probability of applied voltage pulses can be described by a Poisson distribution, where the voltage amplitude and pulse width are taken into account. The randomness is predictable and the distribution function for a set of *N* voltage pulses (neural activity level) with a voltage pulse amplitude *V* can be written as^40^

1 $$ f_{N} = \frac{1}{{1 + e^{{ - d\left( {V - V_{0} } \right)}} }}, $$

where *V*_*0*_ is the threshold voltage at which the probability *f*_*N*_ is equal to 0.5 and *d* is a measure of the slope of the distribution function and therefore of the switching variability. Thus, the steeper the slope, the larger the absolute value of the parameter *d*, and the smaller is the switching window *ΔV*_*sw*_ in which a stochastic encoding of analogue data is possible. The switching window is defined as the voltage range in which the switching probability ${f}_{N}$ is between 2 and 98%.

**Figure 1:**
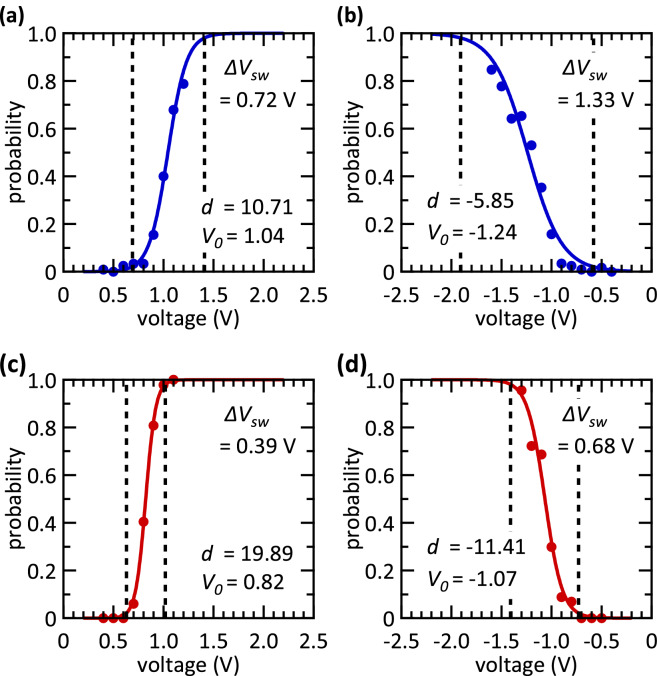
Switching probability of polycrystalline and amorphous devices dependent on the applied voltage. The dots are measured data points and the solid lines are fits of Eq. (1). The parameters of the fits, i.e. *d* and *V*_*0*_, are given in the plots. Furthermore, the size of the switching windows *ΔV*_*sw*_ is given. In (**a**, **b**) the set and reset behaviour of the polycrystalline devices are shown, respectively. The same is depicted in (**c**, **d**) for the amorphous devices. The switching probabilities are determined by measuring 128 polycrystalline and 128 amorphous devices with read-out and switching times of 10 µs.

The device-to-device (D2D) variability of 128 1T-1R devices is evaluated by applying single voltage pulses in the set and reset regime. The switching probabilities, and thus the switching windows *ΔV*_*sw*_*,* as function of single voltage amplitudes of two different types of RRAM cells are illustrated in Fig. 1. As shown in Fig. 1(a, b) the D2D variability of the polycrystalline HfO_2−x_ based devices is larger than the one for amorphous hafnium oxide layers (Fig. 1(c, d)). The grain boundaries of the polycrystalline HfO_2−x_ films are causing a large device-to-device variability^63^. To set the devices from their inertial HRS to the LRS, a positive voltage pulse is applied to the top electrode (Fig. 1(a, c)), while a negative voltage pulse is used to reset the devices back to the HRS (Fig. 1(b, d)). The resistance states are measured at a read voltage of 0.2 V. A threshold current of 20 µA has to be exceeded for a successful set operation, while the read-out current has to be lower than 5 µA to ensure a successful reset operation. The measured data are depicted as dots in Fig. 1, while solid lines represent the distribution function according to Eq. 1, which contains the parameters *d* and *V*_*0*_. The switching windows *ΔV*_*sw*_ are given in Fig. 1 as well. Since the switching processes are based on ion hopping and diffusion, they are stochastically by nature^39,64^. Thus, also a variability occurs between different cycles on one and the same device. This cycle-to-cycle (C2C) variability is shown to differ not significantly from the D2D variability in similar devices^64^. Furthermore, the switching voltages measured here show no correlation with the position of the devices within the 4 kbit array. Thus, using the D2D variability as a measure for stochastic switching is reasonable.

The probability function of the set operation is equal to 0.5 at *V*_*0*_ = 1.04 V for polycrystalline devices, and at *V*_*0*_ = 0.82 V for amorphous devices. For the reset transition, this value is obtained at *V*_*0*_ = − 1.24 V for the polycrystalline devices, and *V*_0_ = − 1.07 V for the amorphous devices. Switching variabilities determined as *d* = 10.71 V^−1^ and *d* = 19.89 V^−1^ are obtained for the set transition for the polycrystalline and amorphous devices, respectively (Fig. 1(a, c)), while for the reset transition *d* = − 5.85 V^−1^ is measured for the polycrystalline devices, and *d* = − 11.41 V^−1^, for the amorphous ones (Fig. 1(b, d)). Thus, the variability in the reset is larger than that of the set process. Consequently, the smallest switching window is observed for the set transition of the amorphous devices (0.39 V), followed by the reset transition of the amorphous devices (0.68 V), the set transition of the polycrystalline devices (0.72 V), and the reset transition of the polycrystalline devices (1.33 V). Hence, the amorphous devices depict a lower variability than the polycrystalline devices for the same switching direction. Additionally, the absolute value of the median switching voltage *V*_*0*_ is lower for the amorphous devices compared to the same switching direction for the polycrystalline devices.

The higher device-to-device and cycle-to-cycle variability of the polycrystalline-HfO_2_ structures might be attributed to the grain boundaries conduction mechanism in polycrystalline-HfO_2_ structures^57^. The higher defect concentration leads to a higher conductivity along the grain boundaries. Furthermore, the cycle stability is affected by thermally activated diffusion of the defects from the grain boundaries. Inversely, the defect concentration in the amorphous hafnium oxide is more homogeneous distributed.

To emulate synaptic plasticity, voltage pulses with amplitudes within the switching windows are applied to the devices. Therefore, the activity *A* of a neuron is encoded in a voltage pulse amplitude according to

2 $$ V = V_{1} + A \cdot \Delta V $$

where

3 $$ A = \frac{N}{\Delta t} $$

here *N* is the number of action potentials arriving at a neuron in the time interval *Δt*, while *V*_*1*_ is the lower bound of the switching window. By optimising *ΔV*, the whole switching window can be exploited to map the activities of the neurons into voltage pulse amplitudes. This allows the mapping of analogue data to the stochastic nature of the binary memristive cells. Therefore, the influence of the switching window range on the learning performance of the network needs to be well understood, and is investigated in depth in the “Results” section of the paper.

### Network structure: stochastic artificial neural network (StochANN)

**Figure 2:**
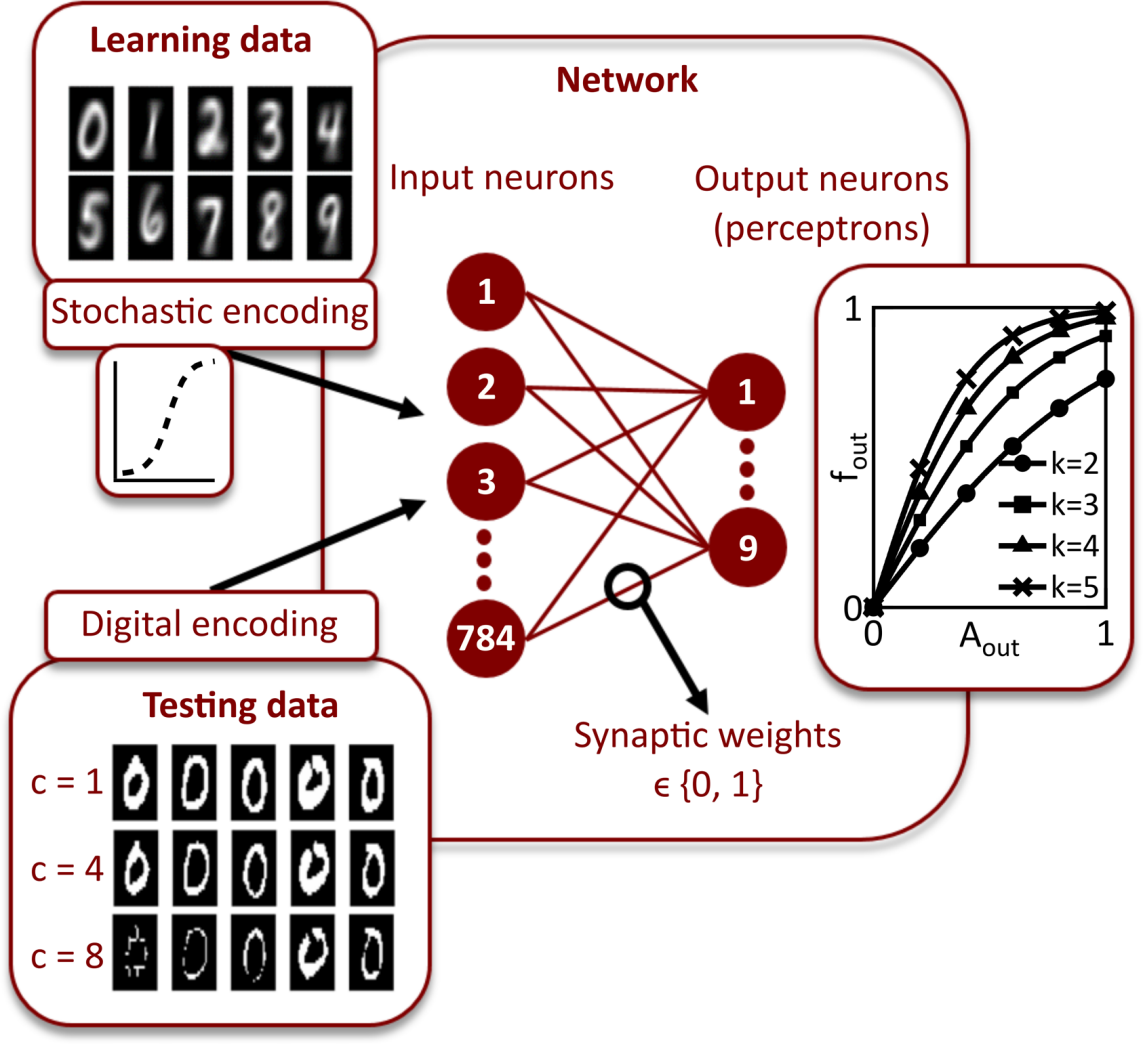
Schematic illustration of the network structure. Each learning data is the average of 100 images from the MNIST data base. The testing data are digitised from the MNIST test data base according to Eq. (5). For learning, the input neurons encode the pixel intensity into a switching probability of the correspondent synaptic weights according to a supervised assignment of the patterns to the receptive fields of the output neurons. For testing, the input neurons map the digitised images to the receptive fields. The output neurons are perceptrons which get activated by the testing data assigning the test images to the respective patterns. The activation function [see Eq. (6)] is depicted for different slopes *k*.

For pattern recognition a two-layer feed forward network is employed, as sketched in Fig. 2. In this configuration, every input layer neuron is connected to every output layer neuron by a memristive device to enable stochastic plasticity according to the computation scheme proposed in Ref.^47^. We want to emphasize, that the learning algorithm exploited in this work is emulating LTP and LTD in biology by implementing a local stochastic learning rule, which differs from conventional learning algorithms for artificial neural networks using the delta rule. For the experimental implementation a mixed-signal circuit board that couples software neurons to hardware synapses is designed. The synapses consist of RRAM devices integrated in a fully CMOS 1T-1R configuration (see “Methods”).

Handwritten digits from the MNIST data base^58^ are used as input patterns. Each learning set consists of 60,000 digits from 250 different writers, and each digit is stored in a 256-level 28 × 28 pixels greyscale image. We use averaged images, which are obtained by combining 100 randomly chosen representations of each pattern and calculating the average greyscale value of the pixels. During learning, the pixel intensities *p*_*i,j*_ of every image *i* are normalised within the interval [0,1] by dividing the values of every pixel *j* by the maximum value *p*_*i,max*_ of the respective image

4 $$p_{i,j,norm} = \frac{{p_{i,j} }}{{p_{i,max} }} .$$

The input images are shown in Fig. 2. These images are rearranged into a 784-row input vector so that each pixel corresponds to one input neuron, while the pixel intensities are encoded by these neurons into switching probabilities. A supervised learning algorithm is employed, where every pattern is assigned to one specific output neuron. The devices that connect the input neurons to the specific output neuron form a receptive field, while the particular resistance values are adjusted during learning. Since binary memristive devices are used, the resistance values are either in the LRS or in the HRS. To enable editing of the grey value images with these binary devices, the normalised pixel intensities *p*_*i,j,norm*_ of the input patterns are encoded into voltage pulses. The amplitudes of these pulses represent the switching probabilities, according to Eq. 1. Either the set or the reset transitions of both technologies are used for learning. To avoid saturation effects, a low amplitude reset pulse is applied to each synaptic device if the learning is done with the set transition, while a low amplitude set pulse is employed if the learning is done with the reset transition. These voltage pulses are applied during each learning iteration.

After learning, the network performance is evaluated using the MNIST test data set containing 10,000 additional digits that differ from the ones previously used. From these data set only 50 representations of each digit are used in the experiment, while the whole data set is exploited in the simulations. Before applying these patterns to the network, their pixels are digitised to 0 or 1 obtaining binary pixel values *p*_*i,j,bin*_. For this purpose, a threshold *Θ*_*i*_ is determined for each test pattern according to:

5 $$ \Theta_{i} = c \cdot p_{i,mean} , $$

where *p*_*i,mean*_ is the mean pixel value of pattern *i* and *c* is a positive constant that regulates the number of bright pixels (Fig. 2, bottom left window). Every test image is applied once to the network. As a result, the pixel intensities encoded by the input neurons are weighted through the receptive fields, which leads to a characteristic activation of the output neurons. The output neurons behaviour is reproduced with a perceptron model that exploits the activation function

6 $$ f_{out} = \frac{{1 - e^{{ - k \cdot A_{out,i,m} }} }}{{1 + e^{{ - k \cdot A_{out,i,m} }} }}, $$

where *k* is a positive constant that defines the slope and *A*_*out,i,m*_ is the normalised activity of the input neurons for the test image *i* weighted by the synaptic connections *w*_*j,m*_ to the output neuron *m* according to

7 $$A_{out,i,m} = \frac{1}{784} \cdot \mathop \sum \limits_{j = 1}^{784} p_{i,j,bin} \cdot w_{j,m} .$$

Therefore, the output neuron whose receptive field best corresponds to the test image shows the highest activation, and associates the test image to the pattern it learned. If several output neurons depict the same pattern, the sums of all activation functions corresponding to the same patterns are evaluated. After all test images are applied to the network, a recognition rate is determined to evaluate the accuracy of the test.

### Investigation of device variabilities

The aforementioned neural computation scheme is inherent to the stochasticity of the memristive devices. The endurance, yield, and retention of the RRAM cells can be used to assess their potential for StochANN. In this context, a closer look at the differences between the polycrystalline and amorphous memristive devices is of great relevance.

**Figure 3:**
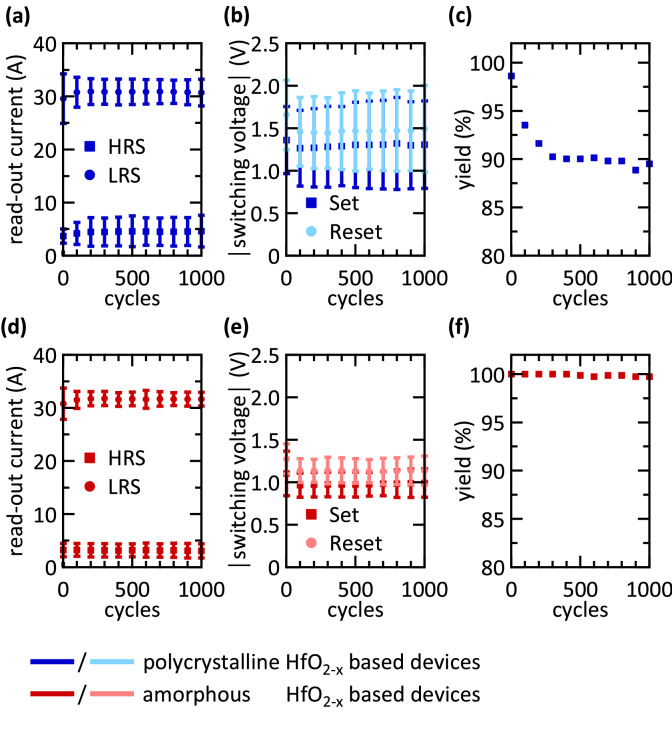
Endurance and yield of polycrystalline and amorphous devices. (**a**, **d**) Show the endurance of the HRS and LRS, (**b**, **e**) depict the evolution of the switching voltages and (**c**, **f**) show the yield of the devices. (**a**–**c**) Correspond to polycrystalline devices, while (**d**–**f**) correspond to amorphous devices. For every data point 128 devices are taken into account. The error bars denote the standard deviation of the averaged measurement data. The read-out and switching times are equal to 10 µs.

Figure 3(a, d) show the evolution of the read-out current over 1,000 switching cycles at a read-out voltage of 0.2 V. The mean values of the HRS and LRS read-out currents remain almost constant over 1,000 cycles, attesting to a high endurance. The standard deviation, illustrated by the error bars, increases during the first 200 cycles for the HRS of the polycrystalline devices. In general, the standard deviation is larger for the polycrystalline devices than for the amorphous devices. Nevertheless, the two resistance states are clearly distinguishable for both types of devices. Figure 3(b, e) present the evolution of the absolute values of the set and reset voltages for both types of devices. Again, the standard deviation of the switching voltages is larger for the polycrystalline devices than for the amorphous devices. For both sets of devices, the mean switching voltages decrease within the first 100 switching cycles and remain constant afterwards.

The yields of the polycrystalline and amorphous devices during 1,000 cycles are shown in Fig. 3(c, f), respectively. Right after electroforming, more than 98% of the polycrystalline and 100% of the amorphous devices are able to switch. The yield of the polycrystalline devices decreases to 93.5% after 100 switching cycles, and to 89.5% after 1,000 switching cycles. In contrast, the yield of the amorphous devices only decreases to 99% after 1,000 switching cycles. For most applications long-term stability is an important factor as it enables the resistance state to be changed and therefore devices to be reused a large number times without encountering device failure. However, this factor is less relevant for the stochastic learning investigated here as it occurs within a few iteration steps. Therefore, both types of devices are well suited for the applications intended here.

**Figure 4:**
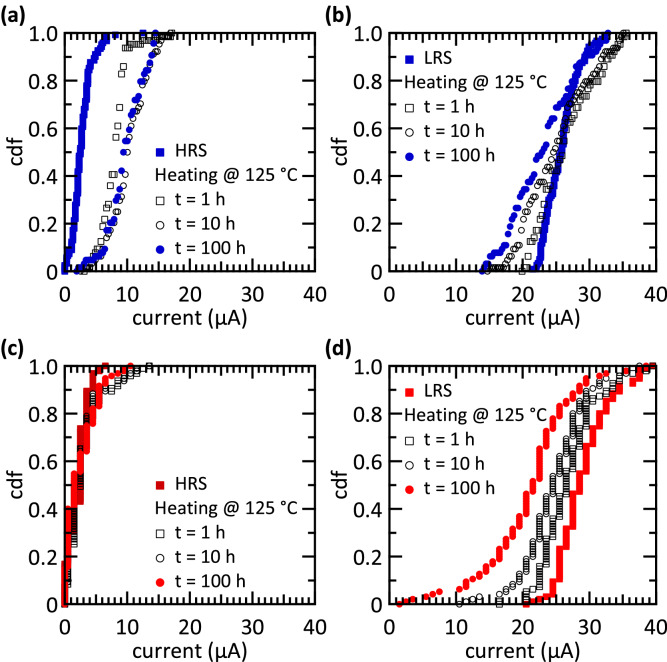
Retention of polycrystalline and amorphous devices. Plots (**a**, **c**) show the retention of the HRS for the polycrystalline and amorphous devices, respectively. In (**b**, **d**) the retention of the LRS is depicted for the polycrystalline and amorphous devices, respectively. The retention is measured for a temperature of 125 °C. Measurements are done with 128 devices for each technology with 10 µs read-out times. The current resolution for (**a**, **b**) is higher than for (**c**, **d**). The more quantised data for the amorphous devices do not show a more quantised retention behaviour.

The retention characteristics of both types of devices are depicted in Fig. 4. The cumulative density function (CDF) of the read-out currents is measured right after reset (HRS) and set (LRS) as well as after 1 h, 10 h, and 100 h. Furthermore, the sample temperature is increased to 125 °C during the investigations. For all current measurements a read-out voltage of 0.2 V is applied.

99% of polycrystalline devices in the HRS (Fig. 4(a)) show a read-out current below 8 µA, whereby 50% of the devices have a read-out current lower than 2.5 µA. After 1 h, the read-out currents increase to a maximum of 15 µA for 98% of the devices, 50% of which display read-out currents lower than 8 µA. This value increases again to 9.5 µA after 100 h. However, these values are much lower than the LRS read-out current of 20 µA. The corresponding values for the LRS are given in Fig. 4(b). Here, read-out currents range from 21.5 to 32.5 µA, while 50% of these values are larger than 25.5 µA. After 1 h, the current range broadens and now goes from 14.5 to 35.5 µA, where 50% of the read-out currents remain larger than 25.5 µA. After 100 h, the read-out currents further decrease. 6.3% of the devices present read-out currents below 15 µA, and the different resistance states are no longer clearly distinguishable for such a small fraction of devices.

The HRS of amorphous devices is particularly stable (Fig. 4(c)). All 128 devices investigated here present read-out currents below 6.5 µA right after reset. After 100 h, 100% of the read-out currents are lower than 10.5 µA. Regarding the LRS, amorphous devices show a relatively strong variation in their read-out currents (Fig. 4(d)). In this resistance state, read-out currents range from 21.5 to 39.5 µA in the beginning, where 50% of the devices present read-out currents below 28.5 µA. After 1 h, the CDF goes from 16.5 to 39.5 µA, and after 100 h the read-out current range broadens further from 2.5 to 38.5 µA. Here, 16% of the devices have read-out currents below 15 µA and 6.3% of those are lower than the critical value of 10.5 µA obtained for the HRS.

Whereas the lifetime of 10 years is required for single memory devices, such a long time is not needed for several neuromorphic circuit concepts^65^. However, long-term retention is a dominant challenge of RRAM devices and is in focus of current research activities. Using HfO_2_/Al_2_O_3_ multilayers instead of HfO_2_ single layers as switching oxide is one prominent technological approach to overcome this issue^66^. The use of refresh cycles during read-out operations represents an algorithmic approach to improve the long-term reliability of RRAM arrays. Also, the training in hardware can be done with devices showing no long-term retention, if the synaptic weights are transferred to devices possessing long-term retention for inference, as it is proposed for DNNs^32^. This way the different requirements for training and inference can be met with different devices.

## Results

Each RRAM chip investigated here presents a total of 4,096 devices. To learn all ten different patterns of the MNIST data set, a higher number of devices would be required (i.e. 7,840). To overcome this aspect, the network is trained with three kind of patterns. To evaluate the network's performance for different patterns, we examine two sets of three patterns each. The first set consists of the patterns “0”, “1” and “9”, while the second set contains the patterns “0”, “3” and “8”. Therefore, the patterns differ more from one another in the former set compared to the patterns in the latter, where the patterns have more pixels in common. Therefore, 3 × 784 = 2,352 individual devices are selected from each type (polycrystalline or amorphous devices) of the 4 kbit chips and are reset to the HRS.

For pattern learning, two types of voltage pulses are required: for the read-out operation of the resistance states, voltage pulses with an amplitude of 0.2 V and a duration of 0.5 ms are applied, while 10 ms pulses with amplitudes determined by the learning rule described above are used for set and reset. The read-out threshold to distinguish between HRS and LRS is set to 10 µA. The slope *k* of the output neurons activation function is equal to 5 [Eq. (6)]. To avoid saturation of the synaptic devices, an additional reset pulse or set pulse corresponding to a 35% switching probability is applied to each device before every learning epoch.

The recognition rates obtained from the digitised test images [*c* = 4 in Eq. (5)] are shown in Table 1 for the different sets of patterns and the two types of devices. Furthermore, the set and reset transitions are employed for learning in order to get a variety of different learning windows *ΔV*_*sw*_ (Fig. 1). If the reset transition is used for learning, the HRS counts as a 1 logical state and the LRS counts as a 0 so that Eq. 7 remains valid.

**Table 1:** Recognition rates of experimental stochastic learning.

Patterns	Switching direction	Polycrystalline	Amorphous
0 3 8	Set	86.3% (± 1.9%)	67.5% (± 5.6%)
0 3 8	Reset	88.5% (± 2.2%)	88.4% (± 5.6%)
0 1 9	Set	89.5% (± 3.1%)	86.5% (± 4.8%)
0 1 9	Reset	75.6% (± 3.5%)	89.9% (± 3.2%)

The recognition rates for both pattern sets determined with both switching directions of the polycrystalline and amorphous devices are shown. Every learning was performed in 5 epochs using the averaged MNIST learning data. Declared are mean values and standard deviations of 5 runs.

**Figure 5:**
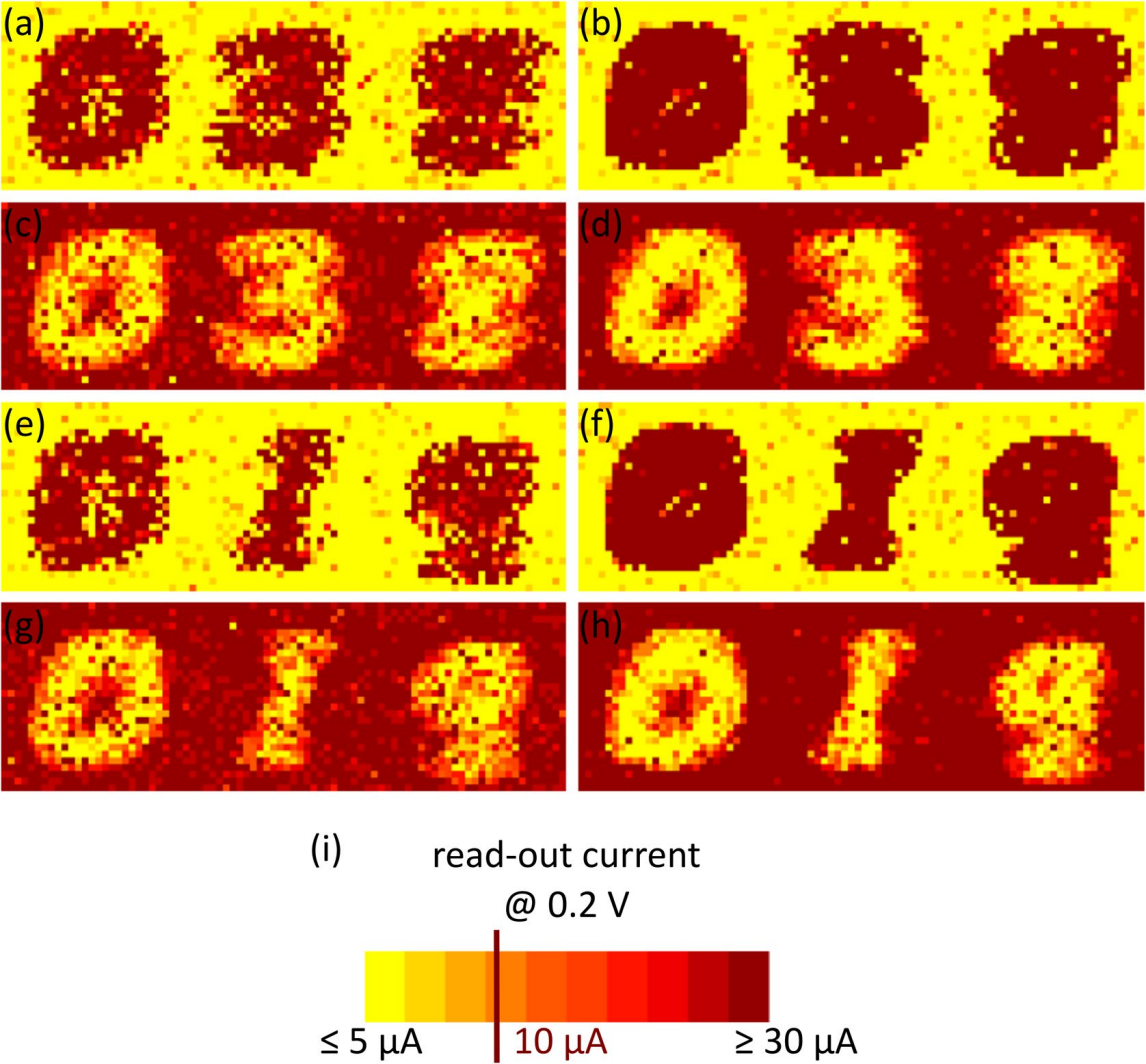
Receptive fields of learned patterns in hardware. The read-out currents of the RRAM devices measured with a voltage pulse *V*_*read*_ with an amplitude of 0.2 V are depicted as two dimensional images. The pixel colour encodes the current of the respective RRAM device. In (**a**–**d**) the learned patterns are {0, 3, 8} using the set transition of the polycrystalline devices (**a**), the set transition of the amorphous devices (**b**), the reset transition of the polycrystalline devices (**c**) as well as the reset transition of the amorphous devices (**d**). In (**e**–**h**) the learned patterns are {0, 1, 9} using the set transition of the polycrystalline devices (**e**), the set transition of the amorphous devices (**f**), the reset transition of the polycrystalline devices (**g**) as well as the reset transition of the amorphous devices (**h**). In (**i**) the encoding of the read-out currents is given.

Figure 5 shows the receptive fields of the two sets of three patterns for both types of devices. The colour of every pixel corresponds to the read-out current of a given RRAM device. This reveals that for both types of devices, the network is able to learn the respective patterns and store them within the resistance values of the devices. Furthermore, we can see that the size of the switching windows *ΔV*_*sw*_ (Fig. 1) affects the learned patterns. For example, the set transition of the amorphous devices results in the narrowest switching window. If this transition is exploited for stochastic learning, the receptive fields become more challenging to differentiate visually (Fig. 5(b, f)) than in the case of the reset transition of the polycrystalline devices, where the switching window is much larger (Fig. 5(c, g)). In particular, the edges here are more graded and thus the patterns are easier to identify with the naked eye, which can be particularly advantageous for cascaded multilayer networks.

**Figure 6:**
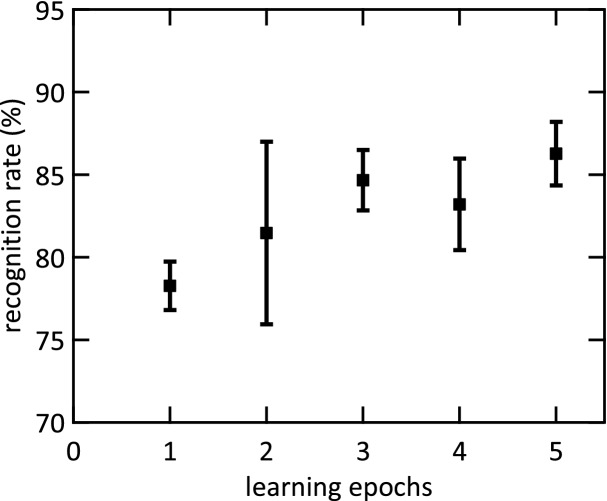
Convergence of the recognition rate. The patterns {0, 3, 8} were learned using the set transition of the polycrystalline devices. The mean values and standard deviations of 5 learning runs are depicted for each data point.

Furthermore, an important feature of the learning algorithm is illustrated in Fig. 6. It shows that the algorithm converges very fast, within five training epochs. This provides considerable advantages in terms of the speed at which the system can adapt to new learning conditions, which could potentially reduce significantly the power consumption of the system.

**Figure 7:**
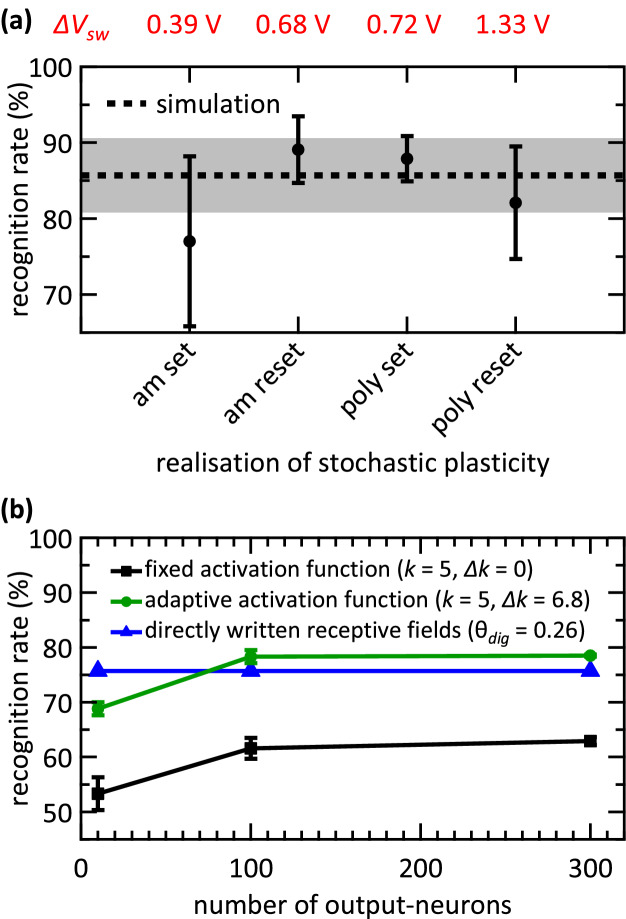
Recognition rates of the StochANN. (**a**) Shows the combination of the experimental results for MNIST subsets {0, 1, 9} and {0, 3, 8}. Depicted are mean values and standard deviations of five experimental runs for each pattern (i.e. ten runs in total for each data point). The mean value of 100 simulation runs for each pattern set is depicted as dashed line while the standard deviation is depicted as grey area, respectively. Five learning epochs are used in every simulation run. While the abbreviations “poly” and “am” denote the polycrystalline and the amorphous HfO_2−x_ based devices, “set” and “reset” denote the transition used to emulate stochastic plasticity. In (**b**) the simulation results for the whole MNIST test set are shown. These results are obtained with a fixed activation function of the output-neurons (black squares) and an adaptive activation function (green circles). The mean values and standard deviations of five runs with five learning epochs are depicted. The direct written receptive fields are shown for comparison (blue triangles). Here, no stochastic mechanisms are involved. Thus, no standard deviation can be denoted. Moreover, an increasing amount of output-neurons does not increase the recognition rate for the latter method, as only one prototype of each pattern can be learned.

Finally, the performance of the network can be quantified by studying the recognition rates. According to Table 1, there is no general correlation between the obtained recognition rates and the size of the switching window. The highest recognition rate for the rather similar patterns {0, 3, 8} is 88.5% ± 2.2% obtained with the reset transition of the polycrystalline devices, which depicts the largest switching window (*ΔV*_*sw*_ = 1.33 V). Moreover, a similar recognition rate (88.4% ± 5.6%) is achieved with the amorphous devices reset transition which presents a much narrower switching window (*ΔV*_*sw*_ = 0.68 V). For the rather different set of patterns {0, 1, 9} the reset transition of amorphous devices (*ΔV*_*sw*_ = 0.68 V) and the set transition of the polycrystalline devices (*ΔV*_*sw*_ = 0.72 V) lead to the highest recognition rates of 89.9% ± 3.2% and 89.5% ± 3.1%. The mean recognition rates combined for both pattern sets are depicted in Fig. 7(a) dependent on the device technology exploited to emulate stochastic plasticity. Here, a correlation between the size of the switching window and the network performance is present. The best recognition rate is obtained with the reset transition of the amorphous devices (89.1% ± 4.4%) followed by the set transition of the polycrystalline devices (87.9% ± 3.0%). Thus, *ΔV*_*sw*_ = 0.68 V and *ΔV*_*sw*_ = 0.72 V for the reset transition of the amorphous devices and the set transition of the polycrystalline devices, respectively, leads to the best recognition results. A wider switching window for the reset transition of the polycrystalline devices (*ΔV*_*sw*_ = 1.33 V) and a narrower switching window of the amorphous devices (*ΔV*_*sw*_ = 0.39 V) results in smaller accuracies of 82.1% ± 7.4% and 77.0% ± 11.2%, respectively. Thus, we show evidence that the device variability has an impact on the network performance. In particular, the size of the switching window must not be too small to optimise the stochastic synapses in the proposed StochANN.

## Discussion

To further assess the performances of the learning scheme with respect to prior investigations, numerical simulations are carried out to determine the theoretical maximum recognition rate. For this purpose, the stochastic learning rule is implemented by generating a random number *r*_*i,j*_ uniformly distributed over the interval (0, 1) for every pixel *j* of every learning pattern *i*. If the pixel intensity *p*_*i,j,norm*_ is larger than *r*_*i,j*_, the respective synaptic connection *w*_*i,j*_ is set to 1. While this relative simple approach does not take into account device variabilities, it provides the limits of the stochastic learning. To reproduce the experimental conditions, every synaptic connection in the learning epoch is additionally set to 0 with a probability of 35% before the stochastic learning rule is applied. This ensures that the synaptic connections will not saturate.

Recognition rates of 84.5% (± 4.6%) for the {0, 3, 8} patterns and 87.0% (± 4.8%) for the {0, 1, 9} patterns are extracted from the simulations. These values are determined after running each pattern set 100 times with five learning epochs. Combining the results of both pattern sets, a recognition rate of 85.7% ± 4.9% is obtained. Although these rates are slightly lower than the maximum values obtained experimentally (Table 1, Fig. 7(a)) they remain within the experimental error margins. Thus, we can conclude that the simulation accurately reproduces the experimental results.

Moreover, the recognition rates are determined for patterns written directly into the synaptic states, thereby ignoring any stochasticity while forming the receptive fields. For this purpose, the input patterns are digitised using a fixed threshold θ_*dig*_. The synaptic connections are set to 1 if the normalised pixels strengths [Eq. (4)] of the input patterns are larger than this threshold, and 0 otherwise. Using thresholds of θ_*dig*_ = 0.15 and θ_*dig*_ = 0.03, accuracies of 94% are obtained for the {0, 3, 8} patterns, and 96.7% for the {0, 1, 9} patterns, respectively.

While the fixed threshold approach leads to higher recognition rates than the stochastic learning scheme, it requires thorough optimisation of θ_*dig*_ over the whole set of patterns. This optimisation becomes more tedious as the number of different input patterns increases. Moreover, variable threshold values for the output neurons are essential to a high recognition performance^34–38,51^ and could further increase the performances of our network. To investigate this aspect, we perform simulations that emulate the learning of all ten patterns of MNIST data set. First, the receptive fields are directly written by digitising the input patterns, and a maximum recognition rate of 75.7% is obtained for a fixed threshold of 0.26. Second, simulations of the proposed network scheme are performed. Recognition rates are determined using the complete MNIST test data set consisting of 10,000 test images. As a result, a recognition rate of 53.3% (± 3.0%) is determined with five learning epochs in five simulation runs. Moreover, an increase in the number of output neurons leads to rates of 61.6% (± 1.9%) and 62.9% (± 0.7%) for 100 and 300 output neurons, respectively. Further increasing the learning epochs and number of output neurons does not lead to any noticeable improvement.

To implement a variable activation function for the output neurons, the slope value *k*_*m*_ [Eq. (6)] of neuron *m* in the output layer is adjusted according to the coupling strength of the connected input neurons after learning:

8 $$ k_{m} = k_{0} - \Delta k \cdot \frac{{\mathop \sum \nolimits_{j = 1}^{784} w_{j,m} }}{784}. $$

here *k*_*0*_ is the base value and *Δk* is a positive constant weighted by the total strength of the synaptic connections. According to this equation, the slope is steeper for neurons that have learned patterns with less active pixels, leading to a stronger activation of those neurons, as it can be seen in Fig. 2. Since the slope adaptation only depends on the final weight distributions, no adaptation during learning is necessary. This leads to recognition rates of 68.8% (± 1.2%), 78.3% (± 1.2%) and 78.5% (± 0.2%) for 10, 100 and 300 output neurons, respectively, determined in five simulation runs with five learning epochs, *k*_*0*_ = 5, and *Δk* = 6.8. The simulation results considering the whole MNIST test set are depicted in Fig. 7(b).

This shows that for stochastic networks with over 100 neurons in the output layer, slightly higher recognition rates are obtained than when the receptive fields are directly written. Furthermore, a detailed examination of the learned receptive fields shows that the stochastic learning scheme leads to more graded fields, which might be of interest for networks with more layers, and results in the slightly improved classification performance.

Previously reported simulations of similar network structures using analogue memristive devices as synapses predict slightly higher recognition rates than the ones obtained in our study^34–37^. In these investigations, unsupervised learning algorithms are employed with different STDPs, and a variety of memristive systems are considered. Devices based on the drift of Ag nanoparticles in a Si layer^2^, or on that of charged defects within an NbO_x_ layer^67^ are explored. With 10 output neurons, these networks achieve recognition rates of 60% for Ag nanoparticles in a Si layer^34,35^, and 65% for NbO_x_ based devices^36^. In the latter, increasing the number of output-neurons to 100 leads to a recognition rate of 82%^36^, while in the former 93.5% of the test images are assigned correctly when 300 output-neurons are employed^34,35^. Incorporating an additional neural layer to realise inhibitory connections between all output-neurons, recognition rates of 82.9% and 95% are achieved with 100 and 6,400 output-neurons and the same amount of inhibitory neurons, respectively^37^. In addition, when a fully connected feature extraction layer is trained using stochastic STDP with 1-bit precision followed by a high performance classification SNN^68^, recognition rates of 93.9% and 95.7% are reached with 1,600 and 6,400 neurons in the feature extraction layer, respectively^51^. In this case, the classification layer has to be trained in the frame domain using stochastic gradient decent (SGD). As input signals, the output of the trained feature extraction layer is needed. After the classification layer is trained, it can be converted to a SNN. The stochastic STDP learning of the feature extraction layer improves the accuracy of the whole network compared to a random weighted feature extraction layer, but the classification SNN is responsible for the high accuracy. Indeed, combining the feature extraction layer with a simpler classifier^69^ leads to a recognition rate of 75.6% using 1,600 neurons in the feature extraction layer^51^. The highest recognition rate reported for a spiking network is 99.1%^70^. However, this high value is reached when the training is done for a convolutional network which is then converted into a spiking convolutional network. Furthermore, a backpropagation algorithm for deep SNNs leads to an accuracy of 98.7% for the MNIST database^71^. A fully hardware-implemented CNN based on multilevel RRAM devices^72^ can achieve recognition accuracies of 96.2%^31^. Here, a five layer network is trained off-line. The weights are then transferred to the eight 128 × 16 1T-1R arrays using two devices as one synapse to obtain positive and negative weights, before re-training the last feature extraction layer on-line. Another mixed-signal approach, in which two analogue RRAM devices^73^ are used as one hardware synapse to obtain positive and negative weights in combination with software neurons, reaches an accuracy of 91.7% using re-scaled MNIST data of 8 × 8 pixel size. Here, a three layer network with one array of 128 × 64 1T-1R devices is utilised with the possibility for on-line learning using SGD^55^. Simulations which incorporate device imperfections show that an extended network with a total of 495,976 devices can achieve an accuracy of 97.3%. The discussed literature is summarised in Table 2. Overviews of the recognition accuracy for MNIST patterns using SNNs, deep SNNs and DNNs, are presented in Refs.^51,68,70,71^.

**Table 2:** Classification accuracy of different artificial neural network approaches on the MNIST test set.

Architecture	Learning rule	Supervised/unsupervised	Realisation with hardware synapses reported	MNIST classification accuracy
StochANN (this work)	Stochastic plasticity	Supervised	For MNIST subset, with memristive devices	78.5% (simulation)
2 layers, feed forward^34,35^	STDP	Unsupervised	No	93.5% (simulation)
2 layers, feed forward^36^	STDP	Unsupervised	For generic patterns, with memristive devices^74^	82% (simulation)
2 layers, excitatory and inhibitory connections^37^	STDP	Unsupervised	No	95% (simulation)
FE layer + classifier SNN^51^	FE layer: stochastic STDP classification layer: SGD + conversion to SNN	FE: Unsupervised CL: Supervised	For Slow Poker DVS dataset, with FPGA	95.68% (simulation)
Spiking convolutional neural network^70^	Backpropagation + conversion to SNN	Supervised	No	99.1% (simulation)
Spiking neural network^71^	Backpropagation for SNN	Supervised	No	98.7% (simulation)
Convolutional neural network^31^	Backpropagation	Supervised	For whole MNIST dataset, with memristive devices	96.2% (experiment)
Deep neural network^55^	SGD	Supervised	For whole MNIST dataset, with memristive devices	91.7% (experiment)

The specified accuracies (last column) are for the whole MNIST test set. Whether these are determined using simulations or experiments exploiting hardware synapses is written in brackets.

In summary, a StochANN based on binary synapses is used to solve the MNIST pattern recognition task. The inherent stochasticity of RRAM devices is exploited to implement stochastic plasticity. This way, analogue data is processed with binary synapses. Direct learning in hardware is enabled with a mature technology using fully CMOS-integrated RRAM devices as synapses and software neurons in a mixed-signal implementation. Two different device technologies, namely devices based on polycrystalline and amorphous HfO_2−x_, are investigated in terms of switching probability, endurance, yield and retention. The devices’ variabilities differ strongly for both technologies. An impact of the switching variability on the network performance is shown in experiments. Furthermore, numerical simulations treating all ten MNIST patterns show promising performances for such a simple network structure.

## Methods

### Sample preparation

The resistive metal–insulator–metal (MIM) cell is composed of sputtered 150 nm thick TiN top and bottom electrode layers, a sputtered Ti layer with a thickness of 7 nm, and an 8 nm HfO_2_ layer grown by Chemical Vapour Deposition (CVD) at 300 °C and 400 °C for the amorphous and the polycrystalline structure, respectively. The devices were integrated in 4 kbit memory arrays organised in a 64 × 64 1T-1R cells configuration. A 1T-1R memory cell consists of an NMOS transistor manufactured in a 0.25 μm CMOS technology whose drain is connected in series to a MIM stack to serve as a selector device. The wordline (WL) voltage applied to the gate of the NMOS transistor allows the definition of the cell current compliance. The area of the MIM resistor is 0.4 μm^2^.

### Electrical characterisation

The electrical properties of the memristive devices were measured in a Cascade PA200 Semi-automatic Probe System, and the current–voltage characteristics were collected with an Active Technologies RIFLE SE system.

### Technical implementation of the network

The algorithm ran on a conventional computer using Visual Studio as programming environment and Visual Basic to simulate the neurons and control the complete experimental setup. The packaged 4 kbit arrays were connected to a printed circuit board (PCB) using a standard 64 pin integrated circuit (IC) socket. The PCB was designed using the software EAGLE developed by CadSoft. A microcontroller (Arduino Mega 2560) was also connected to the PCB to serve the address pins of the RRAM array. The read-out and switching pulses were applied using an Agilent E5263A source measurement unit. The voltage amplitudes corresponding to the switching variabilities were stored in a lookup table with 10 mV resolution.

The numerical simulations of the learning algorithm were done with the software Scilab (version 5.5.2) on a conventional computer.
